# Climate change impact on development rates of the codling moth (*Cydia pomonella* L.) in the Wielkopolska region, Poland

**DOI:** 10.1007/s00484-012-0531-0

**Published:** 2012-02-29

**Authors:** Radosław Juszczak, Leszek Kuchar, Jacek Leśny, Janusz Olejnik

**Affiliations:** 1 Meteorology Department, Poznan University of Life Sciences, Piatkowska 94, 60649 Poznan, Poland; 2 Institute for Agricultural and Forest Environment, Polish Academy of Science, Bukowska 19, 60809 Poznan, Poland; 3 Department of Mathematics, University of Environmental and Life Sciences, Grunwaldzka 53, 50357 Wroclaw, Poland; 4 Department of Matter and Energy Fluxes, Global Change Research Center AS CR, v.v.i., Brno, Czech Republic

**Keywords:** Climate change, Degree days, Codling moth (*Cydia pomonella* L.), Weather generator, WGENK

## Abstract

The main goal of this paper is to estimate how the observed and predicted climate changes may affect the development rates and emergence of the codling moth in the southern part of the Wielkopolska region in Poland. In order to simulate the future climate conditions one of the most frequently used A1B SRES scenarios and two different IPCC climate models (HadCM3 and GISS modelE) are considered. A daily weather generator (WGENK) was used to generate temperature values for present and future climate conditions (time horizons 2020–2040 and 2040–2060). Based on the generated data set, the degree-days values were then calculated and the emergence dates of the codling moth at key stages were estimated basing on the defined thresholds. Our analyses showed that the average air surface temperature in the Wielkopolska region may increase from 2.8°C (according to GISS modelE) even up to 3.3°C (HadCM3) in the period of 2040–2060. With the warming climate conditions the cumulated degree-days values may increase at a rate of about 142 *DD* per decade when the low temperature threshold (*T*_*low*_) of 0°C is considered and 91 *DD* per decade when *T*_*low*_ = 10°C. The key developmental stages of the codling moth may occur much earlier in the future climate conditions than currently, at a rate of about 3.8–6.8 days per decade, depending on the considered GCM model and the pest developmental stage. The fastest changes may be observed in the emergence dates of 95% of larvae of the second codling moth generation. This could increase the emergence probability of the pest third generation that has not currently occurred in Poland.

## Introduction

Insects are the most diverse class of organisms on the Earth. More and more studies are focused on the potential impact of the climate changes on insects because they have, in most cases, detrimental effects on human beings and natural ecosystems (e.g. Harrington et al. 2001). The climate is the dominant factor determining the abundance and distribution of most insect species (Sutherst 2000). Their habitats and survival strategies are dependent on the local weather conditions. Moreover, they are very sensitive to temperature changes, as they are cold-blooded (e.g. Edelson and Magaro 1988; Gilbert and Ragworth 1996; Harrington et al. 2001). The development rates of insects are strongly nonlinear functions of temperature (Logan et al. 2006). Within the bounds of the insects linear response to temperature, they may respond to higher temperature with increased development rates and shorter timing between their developmental stages and consecutive generations (Rosenzweig et al. 2001; Logan et al. 2006). However, temperatures above the optimum temperature of development reduce insect longevity (Rosenzweig et al. 2001) and usually lead to a rapid decrease of their developmental rate and consequently increase insect mortality (Riedl 1983).

The global average temperature has already increased by 0.74°C over the last hundred years (1906–2005). The global linear warming trend (of 0.13°C per decade) over 50 years from 1956 to 2005 is nearly twice as high as it was for the period from 1906 to 2005 (IPCC, Climate Change Synthesis Report 2007). However, in Poland the average air temperature changes were even higher and temperature increased by 0.25°C per decade within the period of 1966–2006. The biggest changes were recorded for the summer period (June–August), when the temperature increased even by 0.33°C per decade (Mager et al. 2009). With the warming climate the cumulative degree-days may increase to 205 *DD* per each 1.0°C average air temperature change, when lower temperature threshold is 0°C and 85 *DD*, when *T*_*low*_ >10°C (Juszczak et al. 2010). According to the IPCC Fourth Assessment Report (WG1) (2007), it is highly probable that at the end of the 21st century the surface temperature will increase from 1.8°C, with a likely range of 1.1–2.9°C for “LOW” SRES scenario, even up to 4.0°C with a likely range of 2.4–6.4°C for “HIGH” SRES scenario. A temperature rise of about 0.2°C per decade is projected for the next two decades for all SRES emission scenarios (IPCC, WG1 Report 2007).

The temperature changes may strongly affect the insects’ physiology and spatial distribution, especially in areas where temperatures tend to be below species optima for most of the year (e.g. Harrington et al. 2001; Yamamura et al. 2006). In these conditions, climate warming may influence insect populations by extending the growing season, altering timing of emergence, increasing growth and development rates, shortening generation times and consequently increasing the number of generations, reducing overwintering mortality and consequently increasing insect populations in the subsequent growing season, increasing the risk of invasion by migrant pests and altering their geographical distribution (Porter et al. 1991; Sutherst 2000; Rosenzweig et al. 2001; Strand 2000; Bergant et al. 2006; Olfert and Weiss 2006; Trnka et al. 2007). Many species have already responded to the warming conditions that occurred over the last century (e.g. Crozier and Dwyer 2006). What is more, the increased frequency of climate extremes can also promote outbreaks of the pest (Gan 2004).

The spatial and temporal distribution of different insects in current and future climate regimes may be predicted by different models, such as CLIMEX (e.g. Rafoss and Saethre 2003) or ECAMON (Trnka et al. 2007). The complexity and main assumptions of models used to simulate the pest development are often different, but most of them are based only on temperature (e.g. Cesaraccio et al. 2001; Logan et al. 2006; Juszczak et al. 2009). To predict the emergence dates of different developmental stages of insects in current conditions, the degree-days models are most commonly applied in the pest management practice. In such cases, developmental rates of invertebrates are often assumed to increase approximately linearly as a function of temperature (e.g. Riedl 1983; Roltsch et al. 1999; Snyder et al. 1999; Bonhomme 2000). However, this assumption may be applied only within the bounds of the linear response to temperature, specific for each individual species, and only for conditions where insects are well adapted to the local climate. In all other cases, the non-linear physiologically based models shall be applied (e.g. Logan et al. 2006), as they take into account the temperature stress induced by environmental temperatures higher than the insect optimum developmental temperature (Maiorano 2011). Nevertheless, the degree-days linear modeling concept is sometimes used to predict the development rates of some insects in future climatic conditions (e.g. Bergant et al. 2006), but the uncertainties of such simulations are very high.

Considering the above, the main goal of this paper was to estimate how the observed and predicted climate changes may affect the development rates and emergence of the codling moth in the southern part of the Wielkopolska region in Poland. In order to simulate the future climate conditions, one of the most frequently used A1B SRES scenario and two different IPCC climate models are considered. Basing on these assumptions, a daily weather generator was used in order to generate temperature values (daily maximum and minimum temperatures) for the future climate conditions. In the paper, degree-days were calculated basing on measured and modeled temperatures in order to present: (1) trends of cumulative degree-days values, and (2) the potential emergence dates of the codling moth at the key developmental stages in the future climate conditions.

The economical importance of the codling moth is related mostly to damage caused by its larvae on apples. This insect can infest up to 90% of an apple crop if orchards are not chemically protected. The codling moth development can be easily controlled if at least a few insecticide sprays are applied on apple orchards during the growing season. Appropriate timing of cover sprays is a key factor in obtaining adequate control of the pest with a minimum insecticide usage (Brunner and Hoyt 1987). This is very important to control the development of the codling moth, as this insect is able to produce, dependending on the climatic zones, up to five generations during one year (Riedl 1983). Pupation of wintered caterpillars begins at an average daily temperature of 10°C, and can last 1–2 months. Mass pupation coincides with flowering of early varieties of apples. Flight of imago occurs in spring at temperatures higher than 16–17°C, and starts to be visible soon after apples' flowering, reaching a maximum in 2–3 weeks during the formation of seed-buds. Moths of the second generation can appear during the flight of the moths of the first generation (Kuznetsov 1994).

In order to approximate the time during which the codling moth reaches a particular development stage throughout the growing season, the degree-days method is most often applied by orchardists. Degree-days, which are necessary for the estimation of the development stages of the codling moth, are most often calculated on the basis of the single sine method with a horizontal cut-off technique (Brunner and Hoyt 1987).

## Materials and method

### Study site

**Fig. 1:**
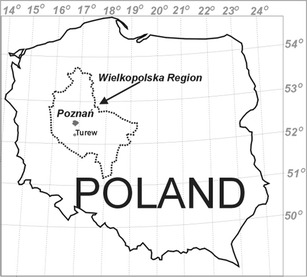
Location of the Turew station, Wielkopolska region, Poland

Analyses were carried out on the basis of the climate data from the research station of the Polish Academy of Science (PAS) in Turew (52°4′0″N, 16°50′0″E), in the south part of the Wielkopolska region, in the middle west of Poland (Fig. 1). The research station belongs to the Institute of Agricultural and Forest Environment of PAS and is located in the middle of the Dezydery Chlapowski Agro-Ecological Landscape Park (Kedziora 2010).

### Weather input data, climate change scenarios and GCM models

All the weather data used in the analyses were collected from the weather station located close to the PAS research station. The set of 34 years of minimum and maximum daily temperatures (from 1972 to 2005) was used to calculate degree-days as well as to generate 500-year series of maximum and minimum daily temperatures for the A1B SRES scenario and for different time horizons.

Among six groups of SRES scenarios discussed in the IPCC’s Fourth Assessment Report (2007a, b), the A1B scenario is most commonly used and best represented in the literature (Nakićenović and Swart 2000). Considering the assumptions of A1B scenario, the global surface temperature will rise until the year 2100 on average by about 2.8°C (in relation to the average baseline temperature 1980–1999) with a likely range of 1.7–4.4°C (IPCC’s Fourth Assessment Report, WG1 2007).

We used the HadCM3 and GISS modelE coupled atmosphere-ocean modeling results to find statistics which were then used to generate air surface temperature for two time horizons: 2020–2040 and 2040–2060. Detailed description of the GISS modelE can be found, e.g. in Schmidt et al. (2006), Aleinov and Schmidt (2006), and Koch et al. (2006). General published references related to the HadCM3 model can be found, e.g. in Gordon et al. (2000), Pope et al. (2000), and Johns et al. (2003). According to predictions based on the GISS modelE (scenario A1B), the average annual temperature in central Europe can increase by 2.8°C (±12%) following a doubling of CO_2_ concentration (ca. in 2040–2060). The average air temperature in winter will increase even by 3.2°C and in summer by 2.0°C (IPCC 2007a, b). The HadCM3 modeling results (based on A1B scenario) indicated that a most probable increase of annual average surface temperature for the north of Poland in the period of 2040–2060 can reach even 3.3°C (±1.1°C). The temperatures in winter and summer may increase up to 3.5°C (ENSAMBLES project, unpublished data).

### Daily temperature generator

The daily maximum and minimum temperatures were generated on the basis of assumptions described above by using the WGENK daily weather generator (Kuchar 2004). The WGENK generator is a modified version of the well-known WGEN generator of Richardson and Wright (1984). The WGEN model has already been tested and well documented for locations in the USA (Richardson and Wright 1984), Alaska (Skiles and Richardson 1998), Europe, Asia (Semenov et al. 1998) and South America (Taulis and Milke 2005). The modified WGENK version, tested in Polish conditions (Kuchar 2004), showed fewer errors for means and variances of the generated data in comparison to the WGEN results.

The procedure used in WGENK for generating daily values of maximum (*T*_max_) and minimum (*T*_min_) temperatures is the same as in WGEN and was described by Richardson (1981) and Richardson and Wright (1984).

### Degree-days calculation

Degree-days values were calculated on the basis of a single-sine method which uses daily minimum and maximum temperatures to produce a sine-wave curve for a 24-hour period, and then estimates a degree-day for that day by calculating the area between the defined temperature thresholds and below the curve (Baskerville and Emin 1969; Allen 1976; De Gaetano and Knapp 1993; Roltsch et al. 1999). The most common single-sine method is recommended to be applied in the field conditions for estimation of degree-days (Pruess 1983). The method assumes that a temperature curve is symmetrical around the maximum temperature. Depending on the considered temperature thresholds (lower and upper) and a place where the sine curve is intercepted by these thresholds, the formulas used for the calculation of degree-days differ significantly (Zalom et al. 1983). If the upper threshold is taken into consideration, then a horizontal cut-off method is usually applied in calculations to subtract the area between the upper threshold and the sine curve, from the area above the lower threshold. This cut-off method assumes that the development of the insect continues at a constant rate at a temperature in excess of the upper threshold and does not increase or stop above this threshold (http://www.ipm.ucdavis.edu/). Formulas used for degree-days calculations are as follows (based on Zalom et al. 1983):

A) If *T*_*max*_ *>T*_*U*_ and *T*_*min*_ *<T*_*low*_ then the sine curve is intercepted by both thresholds and

$$ DD = \frac{1}{\pi }\left\{ {\left( {\frac{{{T_{{ \max }}} + {T_{min}}}}{2} - {T_{low}}} \right)({\theta_2} - {\theta_1}) + \alpha \left[ {\cos ({\theta_1}) - \cos ({\theta_2})} \right] + ({T_U} - {T_{low}})\left( {\frac{\pi }{2} - {\theta_2}} \right)} \right\}, $$

where:

$$ \begin{array}{*{20}{c}}
  {{{\theta }_{1}} = {{{\sin }}^{{ - 1}}}\left[ {\left( {{{T}_{{low}}} - \frac{{{{T}_{{max}}} + {{T}_{{min}}}}}{2}} \right) \div \alpha } \right],} \\
  {{{\theta }_{2}} = {{{\sin }}^{{ - 1}}}\left[ {\left( {{{T}_{U}} - \frac{{{{T}_{{max}}} + {{T}_{{min}}}}}{2}} \right) \div \alpha } \right]{\rm{and}} \propto  = \frac{{{{T}_{{max}}} - {{T}_{{min}}}}}{2}} \\
\end{array} $$

B) If *T*_*max*_ *> T*_*U*_ *, T*_*min*_ *> T*_*low*_ then the sine curve is intercepted by the upper threshold and

$$ DD = \frac{1}{\pi }\left\{ {\left( {\frac{{{T_{{ \max }}} + {T_{min}}}}{2} - {T_{Low}}} \right)\left( {{\theta_2} + \frac{\pi }{2}} \right) + ({T_U} - {T_{low}})\left( {\frac{\pi }{2} - {\theta_2}} \right) - [ \propto \cos ({\theta_2})]} \right\}, $$

where:

$$ {\theta_2} = {\sin^{ - 1}}\left[ {\left( {{T_U} - \frac{{{T_{max}} + {T_{min}}}}{2}} \right) \div \alpha } \right] $$

C) If *T*_*max*_ *<T*_*U*_ *, T*_*min*_ *<T*_*low*_ then the sine curve is intercepted by the lower threshold and

$$ DD = \left\{ {\left( {\frac{{{T_{\max }}{T_{min}}}}{2} - {T_{low}}} \right)\left( {\frac{\pi }{2} - {\theta_1}} \right) + \propto \cos ({\theta_1})} \right\}, $$

where:

$$ {\theta_1} = {\sin^{ - 1}}\left[ {\left( {{T_{low}} - \frac{{{T_{max}} + {T_{min}}}}{2}} \right) \div \alpha } \right] $$

D) If *T*_*max*_ *<T*_*U*_ *, T*_*min*_ *>T*_*low*_ then the sine curve is between both thresholds and

$$ DD = \frac{{{T_{max}} + {T_{min}}}}{2} - {T_{low}} $$

E) If *T*_*max*_ *>T*_*U*_ *, T*_*min*_ *<T*_*low*_ then the sine curve is above both thresholds and

$$ DD = {T_U} + {T_{low}} $$

F) If *T*_*max*_ *<T*_*low*_ *, T*_*min*_ *<T*_*low*_ then the sine curve is below both thresholds and

$$ DD = 0 $$

Degree-days were calculated for two defined lower temperature thresholds of 0.0°C and 10.0°C to demonstrate the changes of *DD* sums above these thresholds in present and future climate conditions. The upper threshold is not considered in these analyses (sine curve is not intercepted by the upper threshold).

### The codling moth (*Cydia pomonella* L.) development model

The developmental thresholds and thermal requirements of different development stages of the codling moth are different, but the base threshold temperature of 10°C is most commonly used in case of assessment of eggs, larvae and pupae development (Glenn 1922; Shel’deshova 1967; Riedel and Croft 1978; Richardson et al. 1982; Howell and Neven 2000; Rafoss and Saethre 2003). A temperature between 15 and 27°C is suggested as optimal temperature for population growth, with 30–34°C as an upper temperature threshold (Howell and Neven 2000). In our analyses we assumed that the upper temperature threshold for the codling moth development equals 31.1°C (Brunner and Hoyt 1981, 1987; Pitcairn et al. 1992). Considering the above-mentioned assumptions we calculated degree-days for each day of the year. The *DD* were cumulated from the beginning of the year and the key degree-days thresholds of 110*DD,* 230*DD*, 650*DD* and 1200*DD* were then found in order to estimate the emergence dates of some key developmental stages of the codling moth (as described in Table 1).

**Table 1:** Codling moth development model. The selected key cumulative *DD* thresholds were set on the basis of Brunner and Hoyt (1981, 1987)

*DD* threshold	Comments
110 *DD*	3-4 adults of the first generation caught in a pheromone trap
250 *DD*	First larvae of the first generation hatch from eggs
650 *DD*	First moths' flights of the second generation
1200 *DD*	Nearly all (>95%) larvae of the second generation are already hatched

## Results

### Temperature changes

**Fig. 2:**
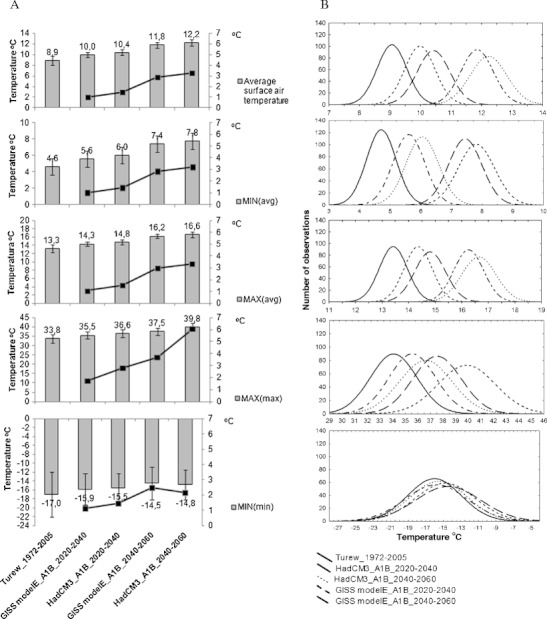
Statistics of the measured and generated temperatures for 500-year data series (**a**), as well as the probability distribution curves for the generated data (**b**), for the present and future climate conditions (SRES scenario A1B) and two IPCC models (GISS modelE, HadCM3) for the two time horizons of 2020–2040 and 2040–2060. MAX represents daily *T*_*max*_, MIN – daily *T*_*min*_ and Average –average temperature; text in the brackets e.g. MIN(max) – these are the highest values from daily *T*_*min*_ over a year; MIN(min) – these are the lowest values from the daily *T*_*min*_ over a year; MIN (avg) – these are the average values from daily *T*_*min*_ for each year of the whole set of 500-year data, etc. Note: The *y*-axis representing temperature values has a different scale, and the supplementary *y*-axis refers to the differences between temperature for each considered generated/modeled temperature parameters and the same temperature parameter for the period of 1972–2005

For each set of 500-years of generated temperatures the average, maximum and minimum values of daily *T*_*max*_, and *T*_*min*_ were calculated for each year. Additionally, the average annual temperature was calculated for each year as an arithmetic average of daily *T*_*max*_, and *T*_*min*_. The variability of all analyzed parameters for all models and time horizons of the measured and generated data is presented in Fig. 2.

The average annual air temperature in Turew station for the period of 1972–2005 equaled 8.9°C. According to the analyzed generated data set, the average annual temperature may increase from 1.0°C (GISS modelE) to 1.5°C (HadCM3) in the period of 2020–2040 and from 2.9°C (GISS modelE) to 3.3°C (HadCM3) in the period of 2040–2060 (with standard deviation ±0.5°C), in relation to baseline average temperature for the period of 1972–2004 (Fig. 2a). The average annual *T*_*max*_ and *T*_*min*_ temperatures may increase more or less to the same extent in the same periods. Much higher changes may occur in the case of daily extreme temperatures. The highest from the daily *T*_*max*_ may increase from 33.8°C (1972–2004) even up to 37.5°C (GISS modelE) and 39.8°C (HadCM3). This means that the highest values of maximum daily *T*_*max*_ recorded over the year may rise even from 3.7°C (GISS modelE) up to 6.0°C (HadCM3) in the period of 2040–2060. At the same time, the maximum daily values of *T*_*min*_ may increase from 3.8°C (GISS modelE) to 6.6°C (HadCM3) for the same period (data not shown). However, much smaller rise of temperature may be expected in the case of minimum values of *T*_*max*_ and *T*_*min*_, although the standard deviation is at least two times higher than in the case of maximum values of *T*_*max*_ and *T*_*min*_ (Fig. 2a).

The variance of predicted average temperatures may slightly increase in the future climate conditions with the increase of average temperatures (Fig. 2b). However, if we focus on extreme temperatures, then we can see that the variance of these temperatures (e.g. MAX(max), MIN(min)) is much larger than that of average values and may increase much more especially for MAX(max) in the future climate conditions. The highest values of daily maximum temperatures (MAX (max)) predicted by the HadCM3 model may be nearly in all cases higher in the future climate conditions (2040–2060) than in present conditions. In each case, there is a noticeable variance of predicted temperatures as a result of HadCM3 modeling. GISS-modelE also predicts the increase of temperatures, but the variance of those temperatures will be smaller than in the case of HadCM3. This could lead to conclusions that with the warming climate, higher values of daily maximum and minimum temperatures, with much larger variance may be expected in central Poland.

**Fig. 3:**
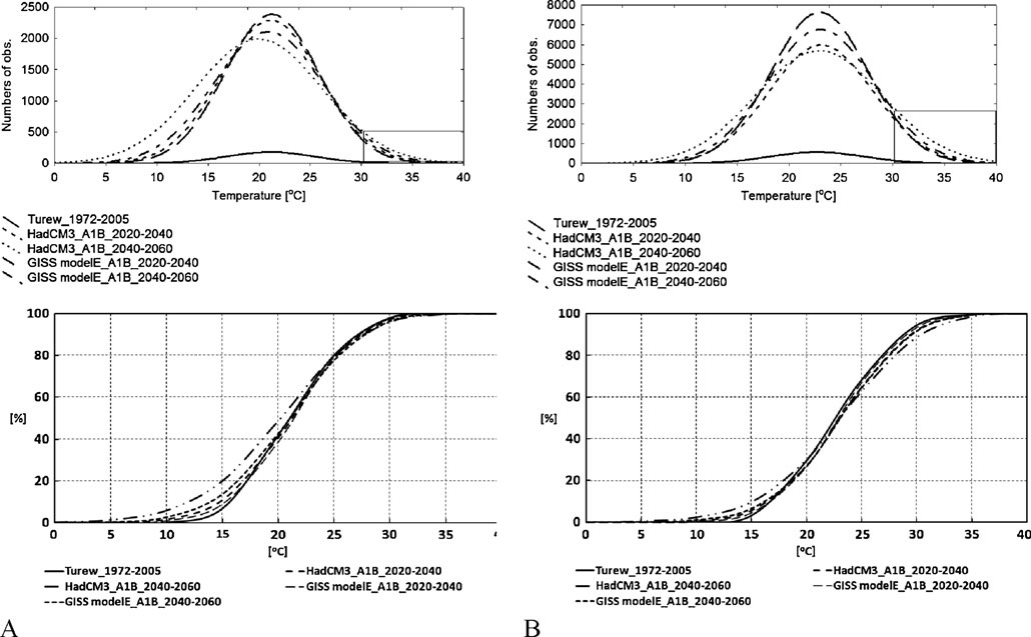
Distribution curves of daily *T*_*max*_ and cumulative distribution curves of daily *T*_*max*_ for the measured and generated datasets and for two periods starting on the days when cumulative degree-days exceeded the 110 *DD* threshold and finished on the day when cumulative *DD* exceeded 250 (**a**) and 650 *DD* (**b**). Measured *T*_*max*_ are restricted to the period 1972–2005, while generated data are related to 500-year datasets for future climate conditions (SRES scenario A1B) and two IPCC models (GISS modelE, HadCM3), for two time horizons of 2020–2040 and 2040–2060

In order to understand how these temperature changes may affect the pest phenology and if it is possible to apply the linear model of the codling moth development in the present and future climate conditions, we analyzed how frequently daily *T*_*max*_ are exceeding the upper temperature threshold of 31.1°C (which may represent a stressful situation for the insect and may reduce or stop its development rate). The analyses were carried out for the periods starting on the day when the 110*DD* threshold was reached and finished on the day when 250 and 650*DD* thresholds (see Table 1) were exceeded (Fig. 3). The analyses were done both for the 34-year set of measured *T*_*max*_ and for the 500-year set of *T*_*max*_ generated for future climate conditions. In the current conditions the threshold temperature of 31.1°C is exceeded by *T*_*max*_ within 1.9% of days of the first period and 5.5% of days of the second period. These numbers may be doubled in the period of 2040–2060 and may reach 4.0% and 11.4% of days of the first and second periods, respectively. Although the numbers of days when the upper temperature threshold is exceeded by daily *T*_*max*_ are likely to increase in future climate conditions, it is rather unlikely that these changes could have detrimental effect on the pest development. One should consider that the upper temperature threshold may be exceeded by *T*_*max*_ for very short time during the day only in the afternoon hours. Taking these assumptions into account we consider that the application of the simple linear *DD* model of the codling moth development is justified in central Poland's conditions and although the model represents some limits it does not lead to incorrect conclusions.

### Changes of cumulated degree-days

**Fig. 4:**
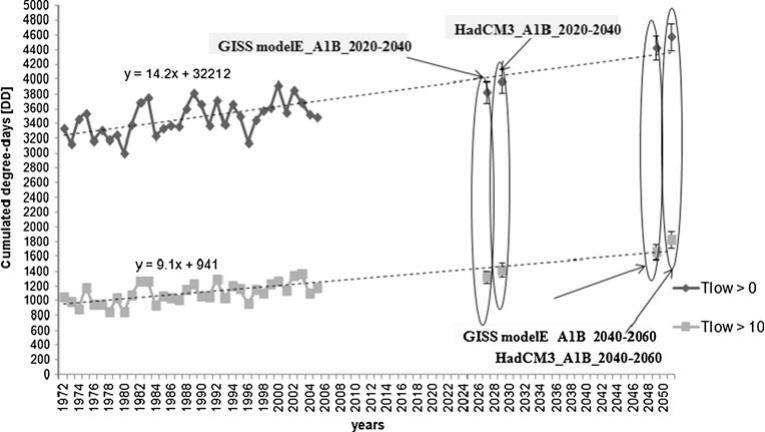
Cumulative degree-days calculated for low temperature thresholds (*T*_*low*_) of 0°C and 10°C based on the measured and generated temperatures for the present and future climate conditions (SRES scenario A1B) and two IPCC models (GISS modelE, HadCM3) for two time horizons of 2020–2040 and 2040–2060

Degree-days (*DD*) were calculated on the basis of measured and generated *T*_*max*_ and *T*_*min*_ for two low temperature thresholds of 0°C and 10°C (without upper threshold limits) in order to find out how the cumulative yearly values of *DD* can change in the future climate conditions (for SRES scenario A1B). Cumulative values of degree-days (*DD*_*cum*_) increased in the period of 1972–2004 (Fig. 4). In the future climate conditions the number of *DD*_*cum*_ calculated above *T*_*low*_ = 0°C can increase from about 3470 *DD* (average for 1972–2005) to 4430 *DD* (GISS modelE) – 4580 *DD* (HadCM3) in the period of 2040–2060. If *T*_*low*_ = 10°C is considered, then the *DD*_*cum*_ can change from 1100 *DD* (1972–2005) to 1670 *DD* (GISS modelE) – 1825 *DD* (HadCM3). The *DD*_*cum*_ calculated based on the data from the HadCM3 model are slightly higher for the same periods than for the GISS modelE. Considering the observed trends, the *DD*_*cum*_ values can increase at a rate of about 142 *DD* per decade when *T*_*low*_ = 0°C is considered, and 91 *DD* per decade when *T*_*low*_ = 10°C.

**Fig. 5:**
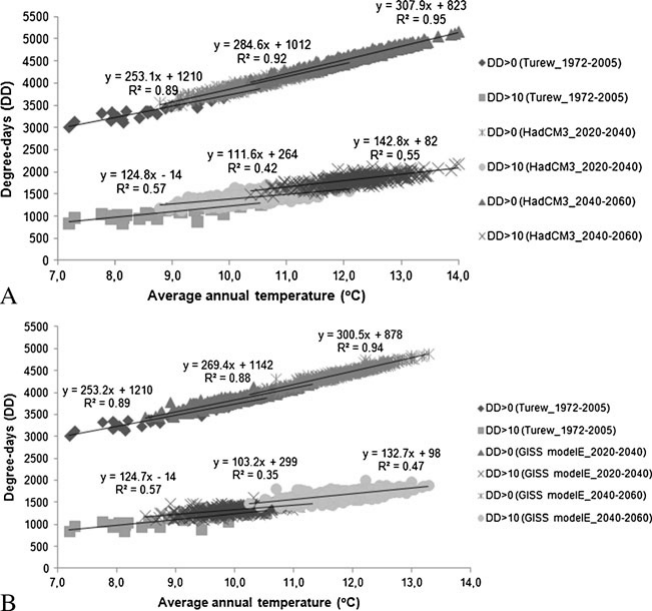
Relationships between the cumulated yearly degree-days and average yearly temperatures. *DD* were calculated on the basis of the generated data above low temperature thresholds of 0°C and 10°C for two time horizons of 2020–2040 and 2040–2060 for models HadCM3 (**a**) and GISS modelE (**b**)

**Fig. 6:**
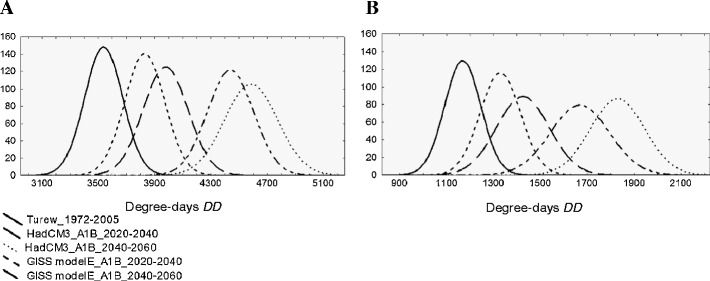
Distribution curves for the modeled yearly cumulated *DD* for low temperature thresholds (*T*_*low*_) of 0°C (**a**) and 10°C (**b**) based on the generated temperatures, for the present and future climate conditions (SRES scenario A1B) and two IPCC models (GISS modelE, HadCM3), for two time horizons of 2020–2040 and 2040–2060

The cumulative degree-days values increase with increasing values of average annual temperatures (Fig. 5). The observed changes are more statistically significant when *DD* are calculated on the basis of HadCM3 modeling results for both temperature thresholds. For higher temperature (later time horizons), the trends are more significant. With increasing values of cumulated degree-days, the variance of such parameters will increase essentially in the future climate conditions and both temperature thresholds (Fig. 6). However, these changes can be more pronounced in the case of *DD* calculated for *T*_*low*_ = 0°C.

### Changes in the emergence dates of key developmental stages of the codling moth (*Cydia pomonella* L.)

**Fig. 7:**
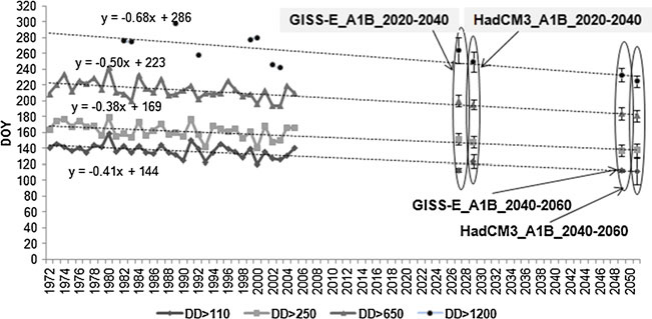
Days of a year when cumulative *DD* exceed the defined thresholds, for the present and future climate conditions (SRES scenario A1B) and two IPCC models (GISS modelE, HadCM3), for two time horizons of 2020–2040 and 2040–2060

The critical dates, when the key developmental stages of the codling moth emerge, may likely occur much earlier in future climate conditions, at a rate of about 3.5–6.3 days earlier per decade than at present (Fig. 7).

**Fig. 8:**
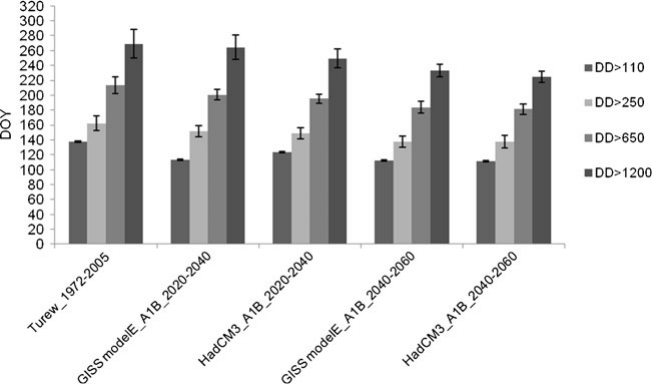
Average number of days when cumulative *DD* exceed the defined threshold for the present and future climate conditions (SRES scenario A1B) and two IPCC models (GISS modelE, HadCM3), for two time horizons of 2020–2040 and 2040–2060

**Fig. 9:**
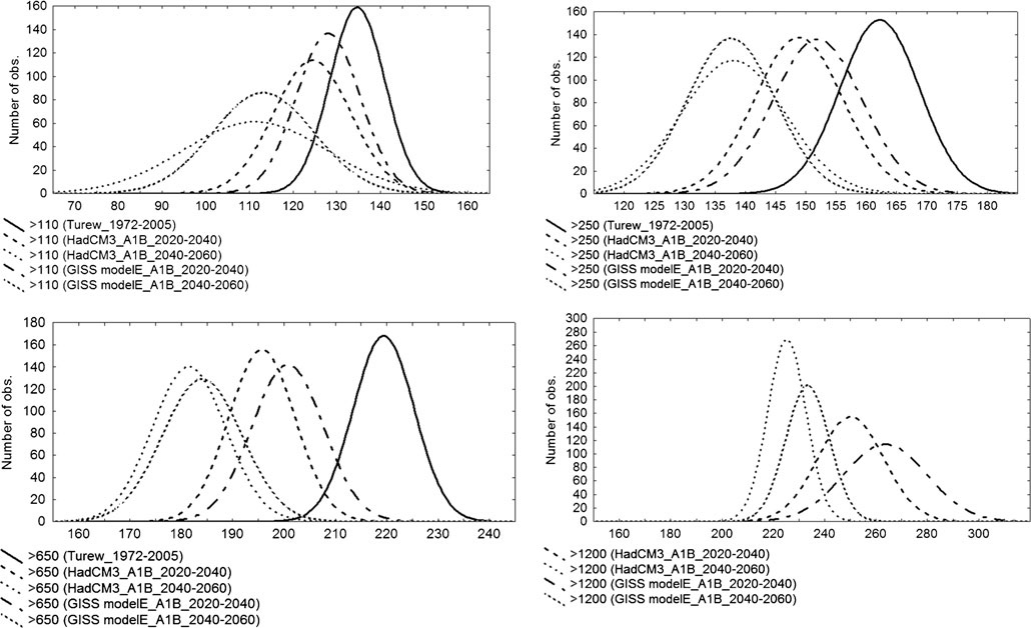
Distribution curves of the dates when the key developmental stages (thresholds >100 *DD*, >250 *DD*, >650*DD*, >1200*DD*) are achieved for the present and future climate conditions (SRES scenario A1B) and two IPCC models (GISS modelE, HadCM3), for two time horizons of 2020–2040 and 2040–2060. Note: The data for the current conditions presented here are generated with WGENK (500-year series) based on the measured Turew data for the period of 1972–2005. The series of data for the threshold of >1200*DD* and current conditions are not presented on the graph, as there were only a few years among 500-year series of data when this threshold was exceeded

The average date of first imago flights, when *DD* exceed 110, occurred at about the 137th day of the year (DOY) in the period of 1972–2005 and it may happen around 110–115 DOY in the period of 2040–2060 (no matter which IPCC model is considered) (Figs. 8, 9). However, with increased temperatures, the variance of the dates when the threshold of 110 *DD* is exceeded will essentially increase in the future climate conditions (Fig. 9). The changes in variance will be much bigger for this key threshold than in the other case. First moths flights of the second generation, when cumulative *DD* reach 650, may appear from 19 days (GISS modelE) even up to 33 days (HadCM3) earlier in the period of 2040–2060 (with no change in variance), than the average for 1972–2005 (214 DOY). What is more important, however, is that the dates when 95% of larvae of the second generation can be hatched (*DD* exceed 1200) may occur from 20 (GISS modelE) to even 44 days earlier at 2040–2060 time horizon, than the average date (269 DOY) in 1972–2005. The variance of dates may also increase with increasing *DD* values, but this change will not be as big as in the case of the first key threshold. This may lead to the scenario in which, as a consequence of warmer conditions and average daily temperatures above 16–17°C, the third generation of the codling moth may occur during the second part of the year in central Poland.

**Fig. 10:**
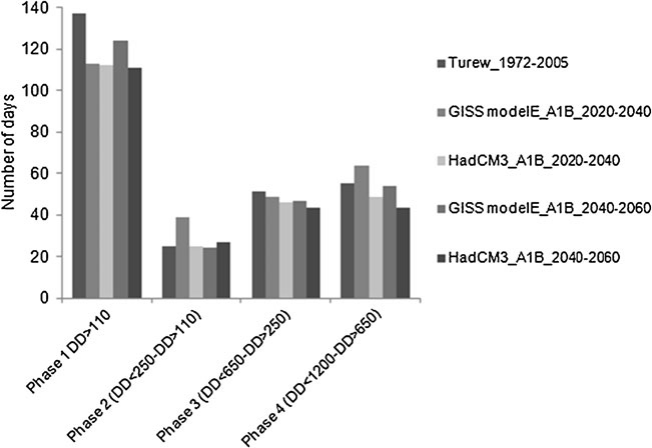
Length of the phases between thresholds for the present and future climate conditions (SRES scenario A1B) and two models (GISS modelE, HadCM3), for two time horizons of 2020–2040 and 2040–2060

On the basis of the received results (Figs. 7 and 8) it can be concluded that the dates of some key developmental stages of the codling moth may appear much earlier in the future climate conditions than at present. The question is however, if with the accelerated occurrence dates of these key stages, the length of phases between these dates may also change. The results of our analyses are shown in Fig. 10. The length of each phase was calculated as a difference between the average date when each defined *DD* threshold was exceeded. The results did not indicate a clear answer to the above question. Generally, it seems that in the future conditions the length of phases between *DD* thresholds may also be shorter than currently. For example, the length of the first phase, when *DD* exceeding 110 is achieved, may last 10–20 days shorter than presently (no matter which model and time horizons are considered). The essential trends are noticeable in the case of third and fourth phases, when the length of the phases shortens with later time horizons (with some exceptions). However, in the case of the fourth phase the interpretation of the data does not give such a clear answer as for the third phase.

## Discussion

The assessment of climate change impacts on agroecosystems and potential adaptation measures at different scales is one of the most important challenges in climate change research (Eitzinger et al. 2009a, b; Eitzinger et al. 2010a, b; Serba et al*.* 2010). Climate change impact on agriculture can have more and more pronounced effects in the future climate conditions. Thus, an early recognition of risks and implementation of adaptation strategies is very important and can be more effective and less costly than forced emergency adaptations (EEA 2007).

In this paper, the authors applied a simple approach based on accumulated degree-days and the climate change scenario in order to assess the change of extreme temperatures, sum of degree-days and emergence time of the codling moth in the future climate conditions. This kind of study has never been performed before for the codling moth in Polish conditions. The combination of a simple *DD* modeling approach, with climate change scenarios and weather generator, is for sure a new concept that gives interesting results for the future theoretical considerations about the potential impact of climate change on the pest development. However, we understand the limitations of such an approach and we know that the application of the linear degree-days model to assess development rate changes of pests in future climates (like it was done, for example, by Bergant et al. 2006) may be disputable, when the conditions to which the model was established will be different. In such conditions, the non-linear physiologically based models should be applied (e.g. Logan et al. 2006), as they may adequately consider the biological processes and their response to the changing climate and the results of modeling may be less uncertain. The criticism of the linear methods may be related to the fact that they may not consider correctly the impact of extreme temperatures higher than optimum temperature on pest development. In the paper we try to indicate that although the frequencies of occurrence of *T*_*max*_ higher than *T*_*U*_ may be even doubled in future conditions (2040–2060), these temperatures may be reached only during few afternoon hours and will not have detrimental effect on the codling moth population. Thus, the application of the linear *DD* model for future climate conditions can be justified, although the uncertainties of such modeling results may be quite high.

The application of the degree-days concept to estimate the timing of pests emergence in current conditions is nothing new and there are a lot of papers where *DD* were used for that purpose. In our paper, the single sine technique with a horizontal cut-off method was applied for the calculation of degree-days following Pruess' (1983) recommendation. Although, there are many other methods of *DD* calculation, which were summarized and compared by Roltsch et al. (1999), the single sine method with horizontal cut-off technique is most commonly used for the estimation of key developmental stages of the codling moth (Brunner and Hoyt 1987). This model is well verified in field conditions (e.g. Pruess 1983; Brunner and Hoyt 1987) for different countries and regions (e.g. Pitcairn et al. 1992).

The weakness of our analyses is that the degree-days thresholds used for modeling were defined for California (USA) conditions and were not experimentally validated in Polish conditions (at least there are not any papers published in recent decades where such information can be found). What is more, the analyses carried out on the basis of the measured data series from 1972–2005 cannot be verified as there is not an easily available source of field experimental data of the codling moth key stages monitoring. The basic monitoring of the codling moth, based on the pheromone traps, is performed in Poland individually by orchardists, a national agency (the Institute of Plant Protection) and research institutes (e.g. Research Institute of Pomology and Floriculture). Results of the monitoring indicated that the first flights of imago occurred in central Poland most often in the middle of May and the number of moths caught in traps was increasing year by year (Plucienik and Olszak 2006). The sources of information related to the codling moth monitoring exist mostly in a non-digital format, which unfortunately does not allow us to make validation of the codling moth model. Nevertheless, considering these limitations, the authors of the paper claim that the performed analyses may indicate the ongoing process of the accelerated development rates of the codling moth in central Poland. This process, which is following the global warming trends, can be more troublesome in the future climate conditions, when this insect can appear in Poland more than one month earlier, giving more than two generations during the growing season. Obviously, the occurrence dates of some key developmental stages of the codling moth can differ from those presented in the paper, but orchardists have to consider these changes in their daily practice by earlier monitoring and exact timing of covering sprays application. The observed trends in central Poland are in line with findings of other authors related to different pests (e.g. Porter et al. 1991; Root et al. 2003; Crozier and Dwyer 2006; Trnka et al. 2007, and many others).

An interesting finding is that, although the emergence of different pest stages may occur much faster in the future climate conditions, the length of the phases between the defined *DD* thresholds will change only insignificantly (although in most cases they can be slightly shorter). Nevertheless, these small changes of the development phases length may lead to the situation when the third generation of the codling moth can occur during the second part of the growing season in central Poland. The question is, however, whether this generation of the pest could have any economical importance, if with longer growing season and warmer conditions the time of apple ripening may also occur earlier during the year. Most probably not, but this issue requires more complementary interdisciplinary studies in the future.

## Conclusions

1. Assuming the A1B SRESS emission scenario, the average air surface temperature in the Wielkopolska region may increase from 2.8°C (according to GISS modelE) even up to 3.3°C (HadCM3) in the period 2040–2060. The biggest changes can be observed in the case of extreme temperatures. The highest from the daily maximum temperatures can increase from 3.7°C to 6.0°C in the period of 2040–2060, depending on GCM’s model. With the warming climate, much higher values of daily maximum and minimum temperatures as well as much higher variance may be expected in central Poland. The probability of heat wave occurrences with extreme *T*_*max*_ values may increase essentially. Whereas frost events with higher values of *T*_*min*_ may be most probably shorter and less troublesome.
2. With the warming climate conditions the cumulated degree-days values may increase at a rate of about 142 *DD* per decade when the low temperature threshold (*T*_*low*_) of 0°C is considered, and 91 *DD* per decade when *T*_*low*_ = 10°C.
3. The key developmental stages of the codling moth can emerge in the future climate conditions much earlier than currently at a rate of about 3.8–6.8 days per decade, depending on the considered GCM model and the pest developmental stage. The fastest changes may be observed in the emergence dates of 95% of larvae of the second codling moth generation. This may increase the emergence probability of the pest third generation, that has not occurred presently in Poland.
4. The length of phases between the key degree-days thresholds may be slightly shorter in the future conditions than presently. The length of the first phase, calculated from the beginning of the year to the day when the first moths are caught in traps, may last even 20 days shorter than presently in the period 2040–2060.
5. There are quite significant differences in modeling results of the analyzed data series of temperatures and degree-days generated on the basis of the considered IPCC models. Temperatures generated basing on HadCM3 modeling results, as well as cumulated degree-days reached higher values and had larger variance in the future climate conditions than the same parameters simulated on the GISS modelE. This indicates some differences between the models themselves, but at the same time the most probable range of predicted changes can be better evaluated. The most important is that both models indicated the same processes and changes but with a slightly different variance.

## Abbreviations

*DD*: Degree-days
*DD*_*cum*_: Cumulative degree-days
*T*_*U*_: Upper temperature threshold
*T*_*low*_: Lower temperature threshold
*T*_*max*_: Maximum daily temperature
*T*_*min*_: Minimum daily temperature
